# First mtDNA Sequences and Body Measurements for *Rattus norvegicus* from the Mediterranean Island of Cyprus

**DOI:** 10.3390/life10080136

**Published:** 2020-08-05

**Authors:** Eleftherios Hadjisterkotis, George Konstantinou, Daria Sanna, Monica Pirastru, Paolo Mereu

**Affiliations:** 1 Agricultural Research Institute, P.O. Box 22016, Nicosia 1516, Cyprus; ehadjisterkotis@ari.gov.cy; 2 Society for the Protection of Natural Heritage and the Biodiversity of Cyprus, Keryneias 6, Geri 2200, Cyprus; fanigeorge@hotmail.com; 3 Department of Biomedical Sciences, University of Sassari, Viale San Pietro 43/b, 07100 Sassari, Italy; pirastru@uniss.it (M.P.); pmereu@uniss.it (P.M.)

**Keywords:** mitochondrial DNA, D-loop, brown rat, alien species, biological invasion

## Abstract

Invasive species are the primary driver of island taxa extinctions and, among them, those belonging to the genus *Rattus* are considered as the most damaging. The presence of black rat (*Rattus rattus*) on Cyprus has long been established, while that of brown rat (*Rattus norvegicus*) is dubious. This study is the first to provide molecular and morphological data to document the occurrence of *R. norvegicus* in the island of Cyprus. A total of 223 black rats and 14 brown rats were collected. Each sample was first taxonomically attributed on the basis of body measurements and cranial observations. Four of the specimens identified as *R. norvegicus* and one identified as *R. rattus* were subjected to molecular characterization in order to corroborate species identification. The analyses of the mitochondrial control region were consistent with morphological data, supporting the taxonomic identification of the samples. At least two maternal molecular lineages for *R. norvegicus* were found in Cyprus. The small number of brown rats collected in the island, as well as the large number of samples of black rats retrieved in the past years might be an indication that the distribution of *R. norvegicus* is still limited into three out of the six districts of Cyprus.

## 1. Introduction

*Rattus rattus* Linnaeus, 1758 (black rat) and *Rattus norvegicus* Berkenhaut, 1769 (brown rat) are considered as the most damaging and successful invasive species on the planet. Due to their commensality with humans they have spread in almost all regions of the world, with *R. rattus* occurring on all continents and *R. norvegicus* excluded only from Antarctica [1,2,3]. Invasive species, and more precisely species of the genus *Rattus,* are known to negatively affect island biota [4,5,6] and are the primary drivers of species extinctions on islands [7,8,9]. Due to the overall dramatic impact of rat invasions on autochthonous biota and on public health, the taxonomic identification of *Rattus* species spreading in specific locations, with particular reference to islands, are of pivotal importance for the conservation of local endemism [10,11]. The genus *Rattus* includes 66 species [3]; therefore, its taxonomy is complex being further complicated by a plethora of synonyms for different species [12]. Identification of individuals, even by experts, is often difficult [13], and for this reason the analysis of morphological traits combined with the genetic characterization of molecular markers is the most efficient system for a well addressed species taxonomic identification [3].

The island of Cyprus is situated at the eastern end of the Mediterranean Sea, south of Turkey, West of Syria, Lebanon and Israel, and North of Egypt, and it has been early and widely colonized by black rats since their spread from the Indian Peninsula [14,15]. On the contrary, the brown rat is not as frequent on the island and the earliest attempt to report species of *Rattus* for Cyprus was by Watson [16,17] who captured through trapping 150 rats from an area near Kyrenia. In addition, 1271 additional specimens were examined from various places along the north coastal region of the island. Watson concluded that “all the rats seen in Cyprus were the variety of brown back and white belly which is usually given the subspecies name of frugivorous” [17]. Nevertheless, Watson also stated that “the brown rat *Rattus norvegicus* also occasionally turns up in the ports but none were encountered elsewhere’. Similar outcomes were obtained by Spitzenberger [18] and Kourtellarides [19] who found *R. rattus* to be common in barn owl (*Tyto alba*) pellets in Cyprus, while no *R. norvegicus* were found in the island. In 1986–1987, one of the authors of the present study (E.H.) performed an extensive trapping of rats in Paphos Forest, an area of 620 km^2^, capturing only *R. rattus* (unpublished data). In 2003, in a list of the mammals of Cyprus, Hadjisterkotis [20,21] listed *R. rattus* and *R. norvegicus* as present on the island. However, the inclusion of *R. norvegicus* in this list was based strictly on oral information from Dr. A. Emanouel of the Central Veterinary Services of Nicosia, who noted that this species was present on the island only at the port of Limassol.

Within the frame of a study on pathogens transmitted by rodents in Cyprus, rats were collected between 2000 and 2003 for the analysis of fleas [22,23]. Authors reported the wide trapping of hundreds of *R. norvegicus* and *R. rattus*, but although fleas were counted and identified using accepted morphologic criteria, no molecular or morphological data were provided for the collected rats.

In 2009, Kryštufek and Vohralík [24] during their visit to Cyprus to study and record the small mammals of Cyprus, captured only black rats, in accordance with the findings reported by Watson [17]. Furthermore, these authors noted that *R. rattus* seems to be by far the most abundant small mammal in Cyprus, particularly so in the maquis and, to a lesser extent, in forests. Since they did not capture any *R. norvegicus* they noted that the presence of this species in Cyprus was dubious. Furthermore, Cucchi et al. [25] performed one of the most extensive live trappings for small mammals in Cyprus, noting the presence of only *R. rattus*, and concluded that this species was introduced with maritime traffic during historical times. From December 2006 to December 2009, the Research Promotion Foundation [26] of Cyprus, financed a project for the qualitative and quantitative study of wild small mammalian fauna in Natura 2000 sites of Cyprus, with the participation of the Wildlife Society of Cyprus, the University of Crete, and the Museum of Natural History of Crete, without finding any *R. norvegicus* on the island. In accordance, in 2016, Nicolaou et al. [27], after 9 years of live trapping of small mammals all over the island, including Limassol port, reported only the presence of *R. rattus*, and stated that “since the species lacks other rodent competitors in Cyprus, they also occur in areas remote from development”. A year later, Nicolaou [28] claimed that “in a number of projects referring to Cyprus, there are references for another rat species, *R. norvegicus*. However, there are no samples from this species until today to excuse its presence, although is possible that existed on the island in the past”. Accordingly, in the same year, Hadjisterkotis [29,30] found only *R. rattus* in the island, and in 2018, Moysi et al. [31] in a study on the diet of the barn owls on Cyprus, did not report any *R. norvegicus*, thus considering their presence on the island as dubious.

During the last few years, several pictures of rats taken by bird watchers in Cyprus appeared on social media. The rats were usually identified as *R. rattus*, and occasionally the same images were reported as *R. norvegicus.* However, according to Kryštufek and Vohralík [24] and Nagorsen [32], while brown rat adults differ clearly from *R. rattus*, more slender juveniles with a pointed muzzle and relatively larger ears can be misidentified as black rats. Relative tail length, however, remains constant regardless of age [24]. Since the age of these rats is unknown, their identification as *R. norvegicus* based only on photographs, may be misleading. Although there are studies presenting body measurements of black rats from Cyprus [24], to the best of our knowledge, there are no molecular and/or morphological characterization of specimens belonging to the species *R. norvegicus* in Cyprus.

In such a context, the primary aim of this study is the assessment of the presence of this species on the island, thus providing the first morphological characterization for individuals of *R. norvegicus* in Cyprus. In order to corroborate the morphological identification, a molecular taxonomic identification was also performed for several specimens, thus providing the first molecular data for this species in the island.

## 2. Materials and Methods

### 2.1. Sample Collection

**Figure 1:**
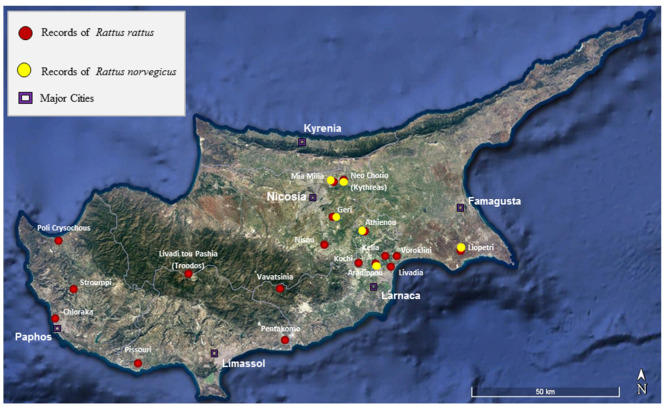
Map indicating the sampling sites in Cyprus where rats were collected.

The number of rats collected and identified for each species is presented in Table 1, and the sampling sites are indicated in Figure 1. Rats were generally collected using snap traps and Herman live traps from January 2019 up to June 2020. Samples were also collected from animals found dead on the road. In addition, 18 skulls of *R. rattus* were collected from five barn owl nests, located at Xylophagou Larnaca, Machaeras Monastery, Kissonerga Paphos, Polis Paphos, and Vavatsinia Larnaca (Table 1).

**Figure 2:**
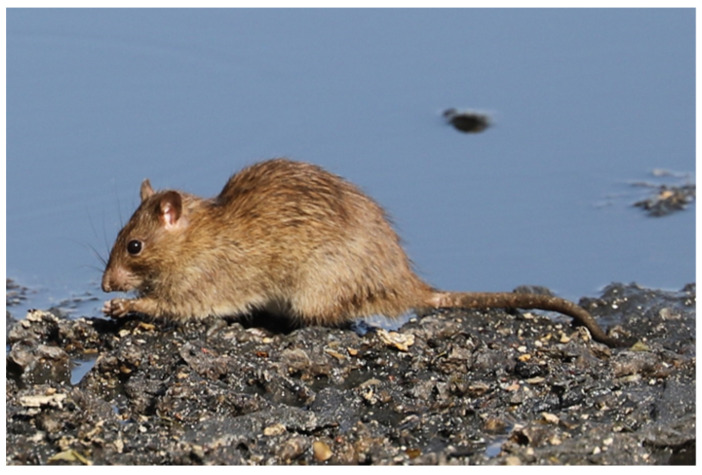
An individual of *Rattus norvegicus* at Mia Milia Sewage Treatment Plant (Photo: E. Hadjisterkotis).

**Figure 3:**
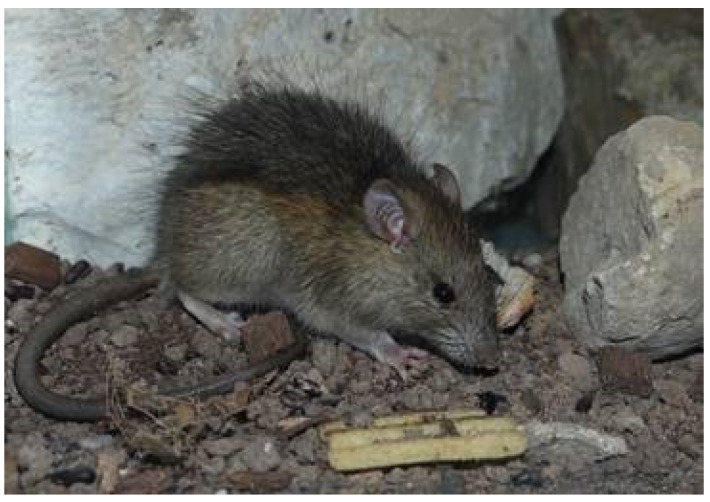
An individual of *Rattus rattus* from Chloraka (Photo: E. Hadjisterkotis).

Black rats were identified from brown rats based on the characteristics descripted by Kryštufek and Vohralík [24], Nagorsen [32], Qumsiyeh [33], and Yiğit et al. [34]. Since in most of the road-killed individuals part of the animal was smashed, one of the characteristics which remained unchanged and allowed instant identification of specimens was the colors of the tail. According to Kryštufek and Vohralík [24], in *R. rattus* the tail is uniformly black, whereas in *R. norvegicus* the tail is indistinctly bicolored, greyish brown above, pale below. For the identification of the species from the skulls found in owl pellets, only complete skulls were used, with intact parietal and interparietal ridges and occipital condyles. A mitochondrial DNA sequencing analysis was carried out in four rats collected between September and December 2019 from three different locations of Cyprus which showed a *R. norvegicus* phenotype (see Table 1 and Figure 1, Figure 2 and Figure 3 for more details) with the aim to support the species identification performed on morphological bases (Table 2). The same molecular analysis was also carried out for one of the samples of *R. rattus* in order to perform a comparative molecular phylogenetic analysis. Pictures of a brown rat and a black rat, respectively, taken in Cyprus during the present study, are shown in Figure 2 and Figure 3.

### 2.2. Examination of Live Specimens and Carcasses

In April 2019, an adult male was collected dead on the road at the center of the village Neo Chorio near Kythrea. A hair sample was collected for molecular analysis (RnC1 in Table 2). The two specimens from Geri, one specimen from Kelia village and one from Geri livestock farms, were skinned in the standard museum manner. From a fifth specimen from Aradippou, the body was damaged and only the head was skinned and preserved. Examination of the stomachs of two specimens from Aradippou and Kelia, indicated that the animals fed on livestock food. These individuals were collected near cow farms. The specimen from Neo Chorio was in an advanced stage of decomposition and after taking body measurements and cleaning the skull, it was disposed. Body measurements (head and body and tail length) were taken using a Tiger 771,588 Vernier Caliper 0–300 mm. For the measurements of the ears and the hind foot, a powerfix digital caliper, version 11/2010 was used. For the weight a portable electronic balance was used, measuring to the nearest gram. For the observation of the color of the tail a Jeweller’s loupe (handheld lens) 30 × 21 mm was used.

One of the authors of the present study (G.K.) for the last few years has been visiting the Mia Milia Sewage Treatment ponds for wildlife photography. He observed that in the ponds there were rats having the characteristics of *R. norvegicus*, as described by Nagorsen [32]. These rats, when compared with *R. rattus* individuals, showed smaller ears and eyes, blunt nose, thick heavy body, and tail shorter than head and body (see Figure 2). G. Konstantinou and E. Hadjisterkotis (authors of this study) visited the area in order to photograph these rats and to visually compare their external characteristics with the rats collected at Neo Chorio and Geri (RnC1 and RnC2 in Table 2, respectively).

Sample identification was based on the following diagnostic external characteristics that allow to distinguish *R. norvegicus* from *R. rattus* [24,32,33,34]:

1. The tail is always shorter than head and body length, indistinctly bicolored, greyish brown above, pale below, ears do not reach the eyes when laid forwards: *R. norvegicus*, Norway rat, brown rat.
2. The tail length is always greater than head and body length, uniformly black. When the ears are drawn forward, they reach the eyes and usually cover them: *R. rattus*, black rat.

**Figure 4:**
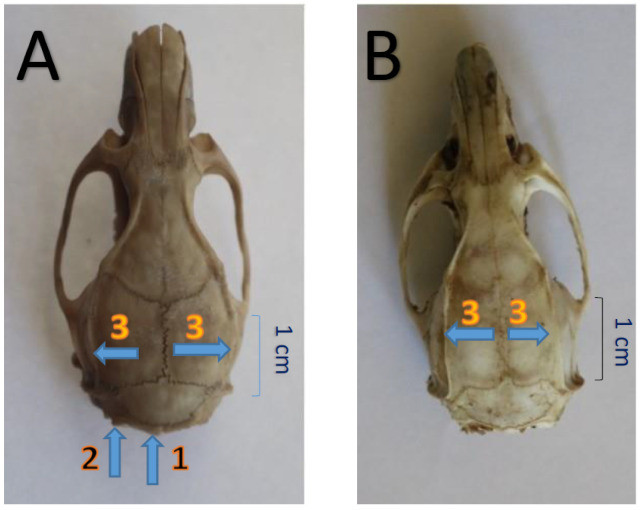
Picture showing the differences in cranial characteristics between the black and brown rats collected in Cyprus. (**A**) The skull of *R. rattus* dorsal view. (**B**) The skull of *R. norvegicus* dorsal view. 1. Supraoccipital ridges, 2. Occipital condyles, 3. Parietal and interparietal ridges.

Species were also recognized based on the diagnostic skull characteristics of *R. norvegicus* and *R. rattus* [24,32,33,34], with particular reference to the shape of the supraorbital ridges. In *R. rattus* supraorbital ridges diverged posteriorly along parietals and close to occipital region they are slightly bent or curved, while in *R. norvegicus* they ran mainly parallel along parietals (Figure 4). According to Pimsai et al. [35] the skull ridges are well-defined in older individuals and characteristic in shape; they are relatively straight and situated close to one another, which gives the impression of a relatively narrow braincase. As it is described by Nagorsen [32], in the Norway rat the braincase is rectangular, temporal ridges straight and nearly parallel, whereas in black rat the braincase is rounded, and the temporal ridges curved.

The palate of *R. norvegicus* is broader behind, bullae are relatively smaller, and the incisive foramen slightly shorter than in *R. rattus*—(see [24]: p. 135, Figure 107 for *R. norvegicus* skull and p. 125, Figure 100 for *R. rattus*). In addition, the skull of *R. rattus* is also more elongated than that of *R. norvegicus* [33] and the occipital condyles are the most posteriorly projecting point of the skull [34].

### 2.3. DNA Extraction, Amplification, and Sequencing

Genomic DNA was extracted from the hairs of five samples (see Table 2 for details) by means of the InstaGene™ Matrix (Bio-Rad) according to the manufacturer’s protocol. Sample quality and DNA concentration were determined via spectrophotometry using a ND-8000 (NanoDrop Technologies, Thermo Fisher Scientific Inc., Wilmington, DE, USA). The DNA mean concentration obtained was 50 ng/μL.

A pair of primers was designed by the authors by means of the “Web Primer: DNA and Purpose Entry” bioinformatic tool available at http://www.candidagenome.org/cgi-bin/compute/web-primer [36] and used to amplify a region of the *R. norvegicus* mitogenome encompassing the first 434 base pairs of the D-loop region (HVS-I domain): Rat_DFw (5′-ccatcaacacccaaagctgat-3′), Rat_DRv (5′-cgagatgtcttatttaagggg-3′).

A standard 50 μL PCR mixture was used, including 250 ng DNA template, 2.5 mM MgCl_2_, 0.20 mM each dNTP, 0.20 μM each primer, 1× PCR buffer, and 2 units Taq DNA Polymerase (Sigma-Aldrich), furthermore, 25 μg of bovine serum albumin (BSA) (5 ng/mL) was also added to the reaction mixture. PCR amplifications were carried out on a GeneAmp PCR System 9700 (Applied Biosystems) under the following conditions: initial denaturation of 95 °C for 5 min, followed by 35 cycles of 95 °C for 50 s, 54 °C (annealing temperature) for 50 s, and 72 °C for 1 min; a final extension of 72 °C for 4 min was also applied. At the end, a post-treatment of 5 min at 72 °C and a final cooling at 4 °C were carried out. Both positive (i.e., a known sample of *Rattus* sp. DNA used to verify that the primers have attached to the DNA strand) and negative controls were used to test the effectiveness of the PCR protocols, and the absence of possible contamination. Electrophoresis was carried out on 2% agarose gels, prepared using 1× SBA buffer (Sodium Boric Acid, pH 8.2) and stained with Gel Red Nucleic Acid Stain (Biotium). PCR products were purified by ExoSAP-IT (USB Corporation) and sequenced for both forward and reverse strands (by means of the same primers used for PCR), using an external sequencing core service (Macrogen The Netherlands). PCRs and sequencing were repeated twice for each sample in order to verify the reliability of results. The PCR products did not show occurrence of aspecificity, excluding the possibility of multiple nuclear mtDNA-like sequences, furthermore, dual peaks of similar height, which could be interpreted as evidence of possible heteroplasmy, were not observed in any of the electropherograms.

#### Dataset Creation, Sequence Comparisons, and Species Identification

The recovered sequences were identified through BLAST analysis in the GenBank nucleotide database (NCBI) (http://www.ncbi.nlm.nih.gov/) in order to estimate the statistical significance of matches and corroborate the taxonomic attribution performed for the specimens on morphological bases.

The amount of genetic variation among *R. norvegicus* sequences, including the number of polymorphic sites (S) and haplotypes (H), the haplotype diversity (Hd), and nucleotide diversity (π), were estimated using DnaSP 6.10.03 [37].

**Figure 5:**
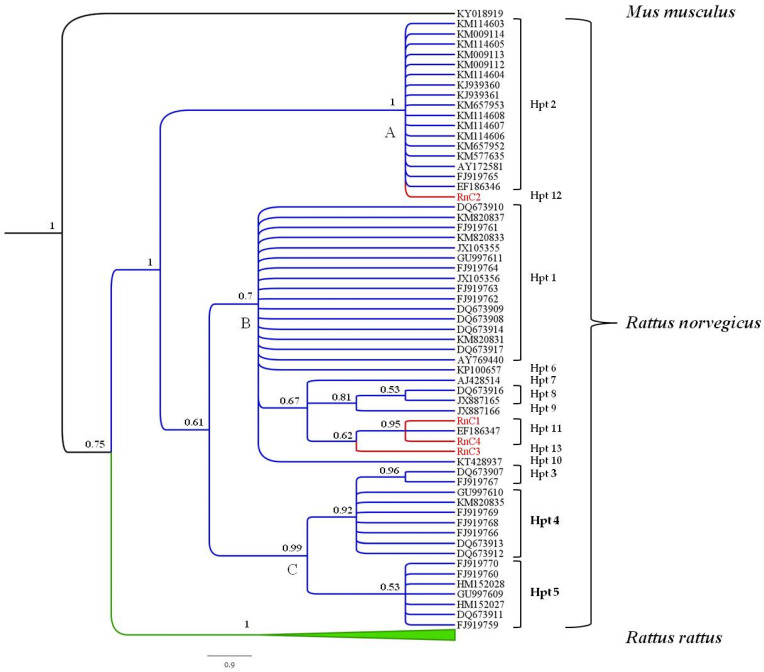
Bayesian phylogenetic rooted tree showing the relationships between the sequences obtained in this study and the mitochondrial D-loop haplotypes available in GenBank for *R. norvegicus* and *R. rattus.* The list of haplotypes grouped within the collapsed clade of *R. rattus* are reported in supplementary Table S1.

Sequences were aligned using the software Clustal X2 [38] and a dataset including the sequences obtained in the present study for *R. norvegicus* (4) and *R. rattus* (1) along with the haplotypes deposited in GenBank for these species which showed the higher levels of similarity at the BLAST analysis (56 for *R. norvegicus* and 39 for *R. rattus*, see Figure 5 for GenBank accession numbers) was constructed.

A phylogenetic tree analysis was performed using the Bayesian Inference (BI) method and a *Mus musculus* sequence (GB # KY018919) was used as outgroup [39]. MEGA 7.0.14 [38] was used to infer the best nucleotide substitution model. The Bayesian tree was inferred using Mr Bayes 3.2.5 [40] via Markov chain Monte Carlo (MCMC). Samples were drawn every 1000 steps over 20,000,000 MCMC steps. The first 10% were discarded as burn-in. Acceptable sampling and convergence to the stationary distribution were checked by inspection of traces using Tracer 1.7 [41] and tree topology was edited in FigTree 1.3.1 (http://tree.bio.ed.ac.uk/software/figtree/).

## 3. Results

### 3.1. Morphological Analysis

The preliminary body measurements, the shape of the skulls, the robustness of the body, and the length and color of the tail of each individual allowed to discriminate between *R. norvegicus* and *R. rattus*, the latter being slimmer with relatively larger ears and a longer black tail (Table 3).

#### 3.1.1. External Characteristics

The number of rats collected and identified for each species is presented in Table 1. In addition, 18 skulls of *R. rattus* were collected from five barn owl nests, located at Xylophagou village of Larnaca (one skull), Machaeras Monastery, Troodos mountains (four skulls), Kissonerga Paphos (six skulls), Polis tis Chrysochous Paphos (two skulls), and Vavatsinia Larnaca (five skulls).

The brown rats were robust and heavily built, compared to slender black rats. The tail length of the brown rats was less than head and body (Table 3), whereas in black rats was longer than head and body.

The color of the brown rats on the back is a mixture of dull ochraceous brown to dark brown and slate grey; it is interspersed by black tips, and occasional white and black hairs. Black hairs are coarser than the soft buff brown hairs. Head, shoulder, and spine are darker; flanks are more buff grey; belly is greyish white of varying intensity. The outer surface of the ears is covered with short, sparse, blackish brown hairs. The inner surface of the ear is naked, pinkish white, with the edges and inner surfaces covered with short, sparse, brownish or whitish hairs. The base of the ear is covered with longer brownish hairs. The upper sides of both the fore and hind feet are covered with tiny whitish hairs, the nails are not pigmented, and the soles of the fore and hind feet are completely naked. When the ear is bend forward towards the eye, is not reaching the eye, or sometimes just reaching the eye. The hairs on the belly, the chest, and the chin are buff white to grey, the bases are greyish to dirty white.

The color of the black rats resembled what Kryštufek and Vohralík [24] considers frugivorous or alexandrines type. In the frugivorous type has pale (white to light grey, yellowish white or pale buff) and sharply defined belly, and dark back which is blackish brown, dark grey brown, reddish brown, or light brown. In the alexandrinus type the back is dark to light brown or grey and belly is darker than in the previous type, albeit clearly paler than back; flanks are frequently grey and demarcation is faint. All possible intermediate stages connect these color types. The tail of the *R. norvegicus* is indistinctively bicolored, due to the tiny dark bristles above and whitish bristles below, which gives the bicolored appearance. The tail bristles of 205 specimens of *R. rattus* examined from the districts Famagusta, Larnaca, Nicosia, Limassol, and Paphos, were all dark, giving the one color appearance.

#### 3.1.2. Cranial Characteristics

The skull of brown rats is considerably robust, the braincase is narrow and elongated. The rostrum is moderately long, and the nasals are rounded off posteriorly. The parietals and interparietal are bordered by well-defined ridges which are straight and almost parallel, which gives the impression of a relatively narrow braincase. The squamosal and maxillary process of the zygomatic arc are laterally widened. The lacrimals project slightly from behind the infraorbital foramen, which is laterally and vertically broadened. The supraoccipital is nearly vertical, and the exoccipital condyles form the most posteriorly projecting point of the skull. In the black rats the skull is strong and heavily built, and supraorbital ridges diverge posteriorly along parietals; close to occipital region they are slightly bent, forming a distinctive rounded braincase. A morphological comparison of the cranial characteristics between black and brown rats is provided in Figure 4.

### 3.2. DNA Analysis

A 483-base pair-long fragment of the hypervariable domain I of the mitochondrial D-loop region from four *R. norvegicus* and one *R. rattus* specimens were sequenced and deposited in GenBank (GB# MN115391-94). A BLAST search for homologous sequences corroborated the taxonomic attribution performed for these individuals on morphological bases. A dataset was constructed including all the haplotypes with higher level of similarity at the BLAST analysis so far available for *R. norvegicus* and *R. rattus* (see Figure 5 for GenBank accession numbers).

The Bayesian tree analysis (Figure 5) provided a topology where the two *Rattus* species formed two well-supported monospecific and monophyletic clades, representing *R. norvegicus* (branches colored in blue in the figure) and *R. rattus* (branches colored in green), respectively (Figure 5).

Three different maternal lineages (A, B, and C, see Figure 5) were detected for the *R. norvegicus* early radiation. Three sequences RnC1, RnC3, and RnC4 grouped within the clade B. RnC1 and RnC4 showed the same haplotype and clustered together with a sequence obtained from a wild brown rat captured in 2007 in French Polynesia (EF186347), while RnC3 showed a haplotype never described before. On the other hand, the Cypriot brown rat RnC2 was phylogenetically related to sequences belonging to the clade A. The Cypriot black rat RrC1 sequence was grouped within the *R. rattus* clade.

Overall, a total of 13 haplotypes, defined by 20 polymorphic sites, were detected for *R. norvegicus* (Table 4). Eight of them were previously reported by Liu et al. 2017 [42] in brown rats from China (D1, D2, D4, D5, D12, D23, D24, D25 in Table 4). The three haplotypes found for the individuals of *R. norvegicus* from Cyprus resulting in nine polymorphic sites and high levels of genetic divergence (*Hd* = 0.833 *π* = 0.011). Estimates of genetic diversity among haplotypes are reported in Table 4.

## 4. Discussion

Identification of individuals belonging to species of *Rattus*, is often difficult [13], and for this reason the analysis of morphological traits combined with the genetic characterization of molecular markers represents the most efficient system for a well addressed species taxonomic identification [3]. Although the morphological criteria used for the first identification of an individual may vary over time, distinguishing among *R. rattus* and *R. norvegicus* is not so difficult, since according to Kryštufek and Vohralík [24], Nagorsen [32], Qumsiyeh [33], and Yiğit et al. [34], as well as our observations in this study, in *R. norvegicus* the tail is always shorter than head and body length, ears do not reach the eyes when laid forwards, and the tail is bicolored. Although Kryštufek and Vohralík [24], notes that the tail of *R. norvegicus* is indistinctly bicolored, grayish brown above, pale below, based on our observations the tail in this species is distinctively bicolored, greyish brown above, whitish below. The distinctive discrepancy with previous descriptions is due to the different color of the tail bristles above and below the tail. On the contrary, in all *R. rattus* individuals the tail bristles above and below were black.

This is the first confirmation based on cranial and body measurements as well as on molecular analysis of the presence of *R. norvegicus* on the island of Cyprus. We also present the first study on the variation of brown rats in Cyprus, providing the first preliminary molecular and morphological data on this alien invasive species, which can become devastating for agriculture and local wildlife. Cyprus is the only center of endemism for birds in Europe [43], a center of endemism for terrestrial mammals [27,29,30], reptiles [44], insects [45,46], terrestrial gastropods [46], and plants [47,48,49,50,51], and represents a biodiversity ‘hotspot’ [20,21,52,53,54]. Biological diversity faces many threats throughout the world, and one of the major threats to native biological diversity is now acknowledged by scientists and governments to be biological invasions caused by alien invasive species such as rats. The impact of invasive species is immense, insidious, and usually irreversible. They may be as damaging to native species and ecosystems on a global scale as the loss and degradation of habitats [53].

The limited number of brown rats so far collected in Cyprus is an indication that the distribution of *R. norvegicus* is limited in the district of Nicosia and Larnaca, around the cities of Aradippou, Geri, Nicosia, and Larnaca, as well as the village of Neo Chorio Kythrea, and the Sewage Treatment Ponds of Mia Milia (Figure 3). In these restricted areas brown rats were found in the sewage treatment plant canals suggesting the possibility that they might spread into new locations following the canals. A similar situation was observed in Israel, where brown rats are more commensal than their relative the black rat but are less widespread. They penetrated human habitations of port cities and managed to reach some cities of the interior. *R. norvegicus* shows to prefer more mesic areas than *R. rattus*. Barrows can be found along stream banks (including sewage streams) in many areas [33].

The ports that are near to the localities where brown rats were collected are the ports of Famagusta and Larnaca, which are about 65 km from Nicosia. Therefore, finding brown rats in the District of Nicosia is an indication that they are spreading toward urban areas where they live around buildings as it was observed in the city of Geri, as well as in the center of Neo Chorio near Kythrea.

The island of Cyprus represents a further case study, where an alien highly invasive species (whose presence on the island was considered as dubious by several researchers), which can be a significant driver of population declines and species extinctions was documented for the first time in the present study. Black rats, according to Hadjisterkotis [29,30], occupy just about every rural habitat in Cyprus. The same was reported by Krystufek and Vohralik [24] who noted that “throughout Turkey and Cyprus, the black rat occupies dwellings (houses, granaries, stables and farm buildings, mills and so forth). Along the Aegean and the Mediterranean coasts of Anatolia and in Cyprus it is abundant and widespread also outside buildings, either in urban and suburban areas (parks, gardens, orchards), in cultivated areas (hedgerows, along fields), and in various types of shrubland, but is most common in humid places and in high maquis”. Hadjisterkotis [55] also observed that black rat is common in forested areas with pine *Pinus brutia* and black pine *Pinus nigra*, as well as golden oak *Quercus alnifolia*, and in torrential river beds on the mountains, where it finds cover in bramble (*Rubus sanctus*), *Smilax asperea*, and myrtle *Myrtus communis.* This species is highly dispersed even on pine trees, where the species uses old nests of woodpigeons as platforms to brake pinecones to extract the seeds [55]. They are excellent climbers and occasionally they prefer to build large squirrel-like nests in dense bramble or even pine trees, and occasionally carob trees (*Ceratonia ciliqua*). Nests, generally at height of 2–3.5 m, are loosely made of leaves and twigs of the available trees or vegetation. The entrance is at one side of the nest, which is flatly domed like a squirrel’s drey, and, from casual inspection, appears to be nothing more than a bunch of dead leaves and twigs, which the thickness of the vegetation keeps in place. In Cyprus during the summer, black rats are a major destruction for carob trees since they eat the fruit and peel the branches, consequently killing the trees. For the first time we also observed them in 2019 and 2020 building roughly round bulky nests on olive trees (*Olea europaea*), by cutting and pilling large numbers of olive tree twigs. In addition, we observed them eating the cambium layer of olive trees, in a similar way that they do with carob trees. Particularly selected for attack are fresh shoots, stripping branches up to 45 mm in diameter. Caches of olives were found under thick vegetation of rock roses (*Cistus* sp.), under stones and wooden boards, in hollow olive trees, even in tunnels under dense grass. Cashes of almonds were found in the hollow branches of almond trees, in abandoned farm buildings, and underneath large rocks (E.H. personal observation).

Black rats are well adapted to small islands, whereas brown rats are mostly observed on the largest islands where humans also occur [56,57,58]. Considering that Cyprus is the third largest island in the Mediterranean, brown rats are expected to adapt and to compete well with black rats thus exerting a stronger impact on native species.

Besides the above reported destruction of trees and crops, rats are particularly harmful in and around farm facilities where they seek food and refuge indoors, gnaw on structural, mechanical, and electrical components, weaken concrete slabs and walkways with their burrowing activities [58]. If not promptly eradicated, they can reach huge numbers, as in the case of a chicken farm in Chloraka, where we trapped 160 black rats.

Rats are reservoirs and vectors of pathogens [55,59,60] that can infect livestock, wildlife species, and humans [58]. A costly disease that was found to infect farm animals and rats, is paratuberculosis that causes significant economic losses for the farm industry with annual estimates of millions of dollars around the world. It was estimated that paratuberculosis costs the U.S. dairy industry alone 200–250 million dollars annually [60]. The etiologic agent *Mycobacterium avium* ssp. *paratuberculosis* (MAP) has been isolated from Cypriot cattle, sheep, and goat populations and dairy food [61,62,63]. Several studies revealed MAP isolations from brown rats [64], foxes, and feral cats [65]. The latter two species are predators of rats on Cyprus, which can become infected by feeding on them. These species may live for several years with home ranges that cover areas large enough to include more than one farm, spreading the disease from farm to farm, and re-infecting farms which eliminated all infected livestock. Therefore, the role of wildlife species (also including both species of rats) in the spreading of MAP to cattle, sheep, and goats must be examined, in order to take the proper management measures for the protection of livestock and humans. Besides the spreading of MAP to cattle, sheep, and goats, epidemiological and clinical data of 193 human cases of murine typhus in Cyprus were recorded and analyzed during a 9-year period (2000–2008). The data collected enhance the belief that murine typhus is a serious public health problem in Cyprus [66].

In the future, extensive studies focused on the demographic patterns of dispersal and habitat selection of *R. norvegicus* in Cyprus, will be needed to assess proper management plans for the protection of public health, agriculture, livestock, and biodiversity, and to depict, if any, the competitive interactions with *R. rattus*.

Furthermore, the expansion of *R. norvegicus* in the island should be also investigated with molecular markers on a large number of individuals to shed light on the phylogeographic structure of this species in Cyprus.

## Figures and Tables

**Table 1:** Sampling plan for the brown rats and black rats collected in the present study. ^+^ visual observation, ^++^ visual observation, dead in the nest of a long-legged Buzzard (*Buteo rufinus*), * road killed, ** captured in snap traps or live traps, ^+++^ skulls collected in barn owl pellets.

	Locality-Village/City and District	*R. rattus*	*R. norvegicus*
Dead specimens	Aradippou-Larnaca	18 *	7 *
	Kochi-Larnaca	1 *	-
	Vavatsinia-Larnaka	14 **	-
	Pentakomo-Larnaka	1 *	-
	Kelia-Larnaka	1 *	-
	Livadia-Larnaka	1 *	-
	Oroklini-Larnaka	1 *	-
	Athienou-Larnaka	-	1 ^++^
	Geri-Nicosia	4 **	3 **
	Nissou-Nicosia	1 *	-
	Neo Chorio Kythreas-Nicosia	-	1 *
	Mia Milia-Nicosia (visual observations only)	-	1 ^+^
	Chloraka-Paphos	160 **	-
	Liopetri-Famagusta	1 *	1 *
	Pissouri-Limassol (Lemesos)	1 *	-
	Troodos (locality Livadi tou Pashia)-Limassol (Lemesos)	1 *	-
	**Total number of collected specimens**	**205**	**14**
Skulls collected from owl pellets	Xylophagou-Larnaca	1 ^+++^	-
	Machaeras Monastery-Troodos mountains	3 ^+++^	-
	Kissonerga-Paphos	5 ^+++^	-
	Polis-Paphos	4 ^+++^	-
	Vavatsinia-Larnaca	5 ^+++^	-
	**Total number of collected specimens and skulls**	**223**	**14**

**Table 2:** Samples selected for molecular analyses. The table reports collection date, code, and sampling location for each specimen whose D-loop sequence was acquired.

Collection Date	Code	Sampling Site	Species
April 2019	RnC1	Neo Chorio-Kythrea	*R. norvegicus*
May 2019	RnC2	Geri-Nicosia	
June 2019	RnC3	Aradippou-Larnaca	
June 2019	RnC4	Geri-Nicosia	
September 2019	RrC1	Koshi-Larnaca	*R. rattus*

**Table 3:** This table reports the morphological diagnostic characters that were considered for the analysis. Body measurements in millimetres of nine *R. norvegicus* from Turkey and 63 *R. rattus* from Turkey and Cyprus, respectively ([24] and references therein), were compared with the specimens from Cyprus here analyzed. The measurements of weight in *R. norvegicus* reported by Kryštufek and Vohralik [24] refer to one specimen only, therefore, no range of values (Min–Max) was provided.

	[24]						Present Study		
	*R. rattus*			*R. norvegicus*					
	N	Mean	Min–Max	N	Mean	Min–Max	N	Mean	Min–Max
Head and body	63	182.7	155–234	9	222.9	192–255	7	195.3	165–230
Tail	60	216.9	180–269	9	187.9	145–216	7	175.4	160–205
Hind foot	40	36.2	33.0–42.0	9	41.6	38.0–44.0	7	40.8	36.5–43.4
Ear	30	23.7	20.0–26.0	9	21.5	20.0–23.0	7	18.5	16.5–18.4
Weight	35	188.4	100–279	1	498	-	5	267	235–285

**Table 4:** Sample distribution and genetic diversity indexes among the clades evidenced in Bayesian phylogenetic tree and among haplotypes. N: number of sequences composing each haplotype; S: number of polymorphic sites; h: number of haplotypes; *Hd*: haplotype diversity; *π*: nucleotide diversity.

Clades	Haplotype	Composition	N	S	h	*Hd*	*π*
A	Hpt-2	KM114603, KM009114, KM114605, KM009113, KM009112, KM114604, KJ939360, KJ939361, KM657953, KM114608, KM114607, KM114606, KM657952, KM577635, AY172581, FJ919765, EF186346	17				
	Hpt-12	RnC2	1				
B	Hpt-1	DQ673910, KM820837, FJ919761, KM820833, JX105355, GU997611, DQ673908, DQ673914, KM820831, DQ673917 (D5), AY769440 FJ919764, JX105356, FJ919763, FJ919762, DQ673909	16				
	Hpt-6	KP100657	1				
	Hpt-7	AJ428514 (D12)	1				
	Hpt-8	DQ673916 (D1), JX887165 (D2)	2				
	Hpt-9	JX887166 (D4)	1				
	Hpt-10	KT428937	1				
	Hpt-11	RnC1, EF186347, RnC4	3				
	Hpt-13	RnC3	1				
C	Hpt-3	DQ673907 (D25), FJ919767	2				
	Hpt-4	GU997610, KM820835, FJ919769, FJ919768, FJ919766, DQ673913, DQ673912 (D24)	7				
	Hpt-5	FJ919770, FJ919760, HM152028, GU997609, HM152027, DQ673911, FJ919759 (D23)	7				
**Total**	**13**		**60**	**20**	**13**	**0.829**	**0.001**

## Supplementary Materials

The following are available online at https://www.mdpi.com/2075-1729/10/8/136/s1, Table S1: *Rattus rattus* sequences retrieved from GenBank and included in the phylogenetic analysis.
