# Supplementary material for: First mtDNA Sequences and Body Measurements for Rattus norvegicus from the Mediterranean Island of Cyprus
Source: Life (Basel). 2020 Aug 5;10(8):136. doi: 10.3390/life10080136 (PMC7460190; doi:10.3390/life10080136)
Supplement: Supplementary file 1 [file life-10-00136-s001.pdf]

Table S1. *Rattus rattus* sequences retrieved from GenBank and included in the phylogenetic analysis.

Species	GenBank Accession number
Rattus rattus	X04735
	GQ891574
	GQ891587
	GQ891571
	KP159555
	KP159542
	KP159541
	KP159533
	KP159525
	KP159551
	KP159545
	KJ603352
	KJ603335
	KJ603326
	KJ603337
	KJ603336
	KJ603317
	KJ603322
	KJ603329
	KJ603332
	KP159529
	KP159549
	EU273707
	MH751490
	MH751493
	EF186354
	EF186357
	LN554990
	LN554991
	LN554996
	LN555005
	HQ588111
	FJ897498
	FJ897499
	FJ897500
	FJ897501
	FJ718279
	KR559037
	KR559039
